# Effects of Endodontic Irrigants on Elastic Modulus and Host-Derived Endogenous Enzymatic Activity of Radicular Dentin

**DOI:** 10.3390/jfb17100486

**Published:** 2026-09-27

**Authors:** Ecehan Hazar, Ahmet Hazar, Roda Seseogullari-Dirihan, Arzu Tezvergil-Mutluay

**Affiliations:** 1 Adhesive Dentistry Research Group, Department of Restorative Dentistry and Cariology, Institute of Dentistry, University of Turku, 20500 Turku, Finland; ahmethazar@beun.edu.tr (A.H.); arztez@utu.fi (A.T.-M.); 2 Department of Endodontics, Faculty of Dentistry, Zonguldak Bulent Ecevit University, Zonguldak 67600, Turkey; 3 Department of Restorative Dentistry, Faculty of Dentistry, Zonguldak Bulent Ecevit University, Zonguldak 67600, Turkey; 4 Department of General Dental Science, School of Dentistry, Marquette University, Milwaukee, WI 53233, USA; roda.seseogullaridirihan@marquette.edu; 5 Turku University Hospital (TYKS), University of Turku, 20500 Turku, Finland

**Keywords:** elastic modulus, endodontic irrigants, maleic acid, matrix metalloproteinases, phytic acid, radicular dentin

## Abstract

Background/Objectives: This study evaluated the effects of endodontic irrigation protocols on the apparent elastic modulus and post-treatment total endogenous matrix metalloproteinase (MMP) activity of mineralized radicular dentin. Methods: Radicular dentin beams obtained from 60 extracted single-rooted teeth were allocated to six groups: distilled water, 2.5% sodium hypochlorite, 17% ethylenediaminetetraacetic acid (EDTA), 7% maleic acid (MA), 1% phytic acid (IP6), and 5% IP6. Apparent elastic modulus was determined before and after immersion using three-point bending, while total MMP activity was quantified using a generic colorimetric assay. Data were analyzed using two-way mixed ANOVA, Welch’s ANOVA with Tamhane’s T2 post hoc test, and Spearman’s correlation (α = 0.05). Results: A significant irrigation protocol × time interaction was observed for apparent elastic modulus (*p* < 0.001), with the greatest reductions following 7% MA and 5% IP6 treatment. Relative total MMP activity differed significantly among the irrigation protocols [Welch’s F(5, 23.05) = 36.285, *p* < 0.001] and was highest following 7% MA and 5% IP6 treatment. No statistically significant difference in relative total MMP activity was detected between the 1% IP6 and 17% EDTA protocols (*p* = 0.086). ΔEM% and relative total MMP activity were negatively correlated in the pooled analysis (r_s_ = −0.679, *p* < 0.001); however, this association was no longer statistically significant after adjustment for irrigation protocol (partial r_s_ = −0.146, *p* = 0.288). Conclusions: Protocol-dependent responses were observed. Low-concentration IP6 warrants further investigation as a potential EDTA alternative, although equivalence and clinical substitutability remain unestablished.

## 1. Introduction

The organic and inorganic composition of radicular dentin is essential to its mechanical integrity and biological function. Radicular dentin consists of approximately 70% inorganic material, mainly hydroxyapatite, and 30% organic components, primarily collagen and non-collagenous proteins [1]. The balance between these components is critical for maintaining dentin’s structural integrity. The inorganic phase contributes to hardness and strength, whereas the organic matrix provides flexibility and toughness. Ideally, an irrigation solution should not adversely affect dentin’s physical or chemical properties. Nevertheless, several studies have shown that various irrigants can alter dentin’s organic and inorganic components, thereby altering its chemical composition and mechanical behavior [2,3,4,5,6,7,8]. Such changes are thought to make endodontically treated teeth more susceptible to fracture [4,9]. Furthermore, modifications to dentin structure induced by irrigants can affect microbial adhesion and the sealing ability of restorative materials [2]. Therefore, selecting irrigating solutions that effectively eliminate the smear layer and microbial remnants while preserving dentin integrity is crucial for successful endodontic outcomes.

Sodium hypochlorite (NaOCl) is the primary irrigant of choice in endodontic therapy owing to its broad-spectrum antimicrobial activity and potent tissue-dissolving properties [7]. However, because NaOCl alone is insufficient to remove the entire smear layer, it is used in conjunction with chelating agents [2,4]. Ethylenediaminetetraacetic acid (EDTA) is widely used at 17% concentration to remove the inorganic portion of the smear layer [2]. Nevertheless, prolonged exposure to strong chelators such as EDTA can alter the biomechanical properties of radicular dentin, decreasing hardness and increasing susceptibility to root fracture [3]. In addition, EDTA is not readily biodegradable, raising concerns about potential extrusion into periapical tissues [10]. These limitations have prompted research into alternative chelating agents that can effectively remove the smear layer without compromising dentin’s mechanical properties. Maleic acid (MA) is a mild organic acid commonly used as an acid conditioner in adhesive dentistry. Compared with EDTA, MA exhibits lower toxicity and greater antimicrobial activity and has been reported to effectively remove the smear layer from the root canal, including the apical third [11]. Because of its pronounced effects on both organic and inorganic components, MA has been proposed as a potential alternative to EDTA as an irrigant [12]. Phytic acid (IP6, inositol hexakisphosphate), proposed by Nassar et al. [10] as an alternative to EDTA, is a naturally occurring phosphate compound abundant in plant seeds and bran and plays a key role in various cellular processes. Due to its multiple negatively charged phosphate groups, IP6 efficiently chelates multivalent cations such as calcium, magnesium, and iron. It has been reported that IP6 effectively removes the smear layer while exhibiting lower cytotoxicity toward pulpal and osteoblast-like cells compared with EDTA, suggesting its potential as a more biocompatible endodontic chelating agent [2].

Matrix metalloproteinases (MMPs) are zinc- and calcium-dependent proteolytic enzymes that degrade extracellular matrix components such as collagen, and their activation may compromise dentin cohesion and mechanical resistance by disrupting the collagen network that contributes to the elastic modulus [13,14]. In coronal dentin, previous studies have shown that acidic agents used in etch-and-rinse or self-etch adhesives, as well as demineralization procedures, can reactivate endogenous MMPs, leading to collagen fibril degradation and reduced bond durability [5,15]. MMP expression and activity have also been detected in radicular dentin, including MMP-2, MMP-8, and MMP-9, which are associated with resin–dentin bond degradation [13,16,17,18]. Accordingly, MMP inhibitors have been suggested to improve the long-term stability of post-core restorations in endodontically treated teeth by suppressing MMP activity [19,20]. However, few reports in the literature have addressed the effects of endodontic irrigation solutions on MMP activity in radicular dentin [18,21]. Understanding this interaction is essential, as alterations in both mechanical and enzymatic properties may influence the long-term prognosis of endodontically treated teeth [6,9,18,21].

To date, no study has evaluated the effect of phytic acid on total MMP activity or the apparent elastic modulus of radicular dentin, and there are no available data regarding the effect of maleic acid on total MMP activity. Therefore, this study aimed to assess the effects of different endodontic irrigation protocols on the apparent elastic modulus and post-treatment total endogenous MMP activity of mineralized radicular dentin. The first null hypothesis was that the tested irrigants would not alter the apparent elastic modulus of mineralized radicular dentin. The second null hypothesis was that post-treatment total endogenous MMP activity would not differ among the tested irrigation protocols. The third null hypothesis was that there would be no significant association between the percentage change in apparent elastic modulus and relative total MMP activity.

## 2. Materials and Methods

Sample-size adequacy for the primary irrigation solution × measurement time interaction in apparent elastic modulus was reassessed using G*Power software (version 3.1.9.7, Heinrich-Heine-Universität Düsseldorf, Düsseldorf, Germany) for a two-way mixed ANOVA (repeated-measures, within–between interaction). An effect size (f) of 0.6044, obtained from the apparent elastic modulus results of a previous laboratory study [22], was used as the best available external estimate of the expected mechanical effect. Using a type I error probability (α) of 0.05, a statistical power (1 − β) of 0.95, six between-subject groups, two within-subject measurement time points, an assumed correlation of 0.50 between repeated measurements, and a nonsphericity correction of 1.0, the minimum required total sample size was calculated as 24 specimens (*n* = 4 per group). Nevertheless, ten specimens per group were included to improve the precision and robustness of the estimates and to account for potential specimen loss or damage during testing, resulting in a total sample size of 60 specimens (*n* = 10 per group). Teeth with radicular caries, resorptive defects, cracks, previous endodontic, restorative, or prosthetic treatment were excluded. Periapical radiographs were taken from each tooth to confirm the presence of a single root canal. The teeth were stored at 4 °C in a 0.9% NaCl solution with 0.02% sodium azide added to prevent bacterial growth until use. Extracted teeth were obtained from anonymous donors. No identifiable patient-related information was used; therefore, formal ethics approval was not required under applicable local regulations (Tissue Act, Section 20). All chemicals, unless otherwise stated, were purchased from Sigma-Aldrich (St. Louis, MO, USA).

### 2.1. Specimen Preparation

The preparation of radicular dentin beams and the subsequent experimental workflow are schematically illustrated in Figure 1. The schematic figure was generated with the assistance of OpenAI image-generation tools and was subsequently reviewed and edited by the authors to ensure consistency with the experimental procedures. The teeth were decoronated 1 mm under the cemento-enamel junction using a low-speed water-cooled saw (Isomet 1000 Precision Saw, Buehler Ltd., IL, USA). A rectangular radicular dentin beam of 0.3 (±0.01) mm thickness, 3 mm width, and 7 mm length was prepared from the coronal region of each root parallel to the long axis of the tooth. The beam was taken from the area closest to the canal lumen. To enable repeated measurements on the same surface, a dimple was created at the corner of each beam. The dentin beams were rinsed in distilled water at 4 °C for 20 min, with continuous stirring at 60 rpm, to remove any surface residue (Loopster digital, IKA, Staufen, Germany). The beams were examined using a stereo microscope (Leica M60, Leica Microsystems, Wetzlar, Germany), confirming the absence of canal lumen, residue, or cementum. One radicular dentin beam was obtained from each tooth, yielding 60 beams from 60 teeth. Accordingly, each tooth, represented by its single beam, constituted one independent experimental unit. The beams were placed in individually labeled 96-well plates.

### 2.2. Apparent Elastic Modulus

The initial apparent elastic modulus of each beam was measured with a three-point bending test using a universal testing machine (Autograph AGS-X, Shimadzu, Kyoto, Japan). A 3-point bending jig (Instron, Instron Inc., Norwood, MA, USA) with a 5 mm span between 2 supports was used. The samples were placed with the created dimple on top and the bearing surface centered on the support. Dentin beams were fully submerged in distilled water during the test. Dentin beams were tested under flexure to 3% strain at a displacement rate of 0.5 mm min−1 using a 5 N load cell (SMT1-5N, Interface, Scottsdale, AZ, USA). The load was returned immediately to 0% stress after reaching 3% strain. Three-point bending at this strain level has been described as a non-destructive procedure suitable for repeated measurements on the same dentin beam [23].

Load–displacement curves were converted to stress–strain curves, and the apparent elastic modulus (*E*) was then calculated using the following equation and expressed in MPa:

$$E={mL}^{3}/4bh^{3},$$

where *m* is the steepest slope of the linear portion of the load–displacement curve recorded before the 3% strain limit (N/mm), *L* is the span length (5 mm), *b* is the width, and *h* is the thickness of the radicular dentin beam in mm [23]. The linear region was operationally defined as the portion of the curve exhibiting the steepest linear slope before the maximum applied strain, and the same criterion was applied to all specimens at both measurement time points. At each measurement time point, each specimen was tested twice, and the mean value was used for analysis. Apparent elastic modulus values calculated using the equation were initially obtained in megapascals (MPa) and divided by 1000 for presentation in gigapascals (GPa).

After baseline apparent elastic modulus measurements, the specimens were ranked according to their initial values and allocated to six equal-sized groups (*n* = 10 per group) using a rank-based baseline-balanced allocation procedure. Specimens were distributed across the groups to minimize differences in mean baseline apparent elastic modulus. No simple randomization, predefined stratification, formal block randomization, or pair matching was performed. Following allocation, the normality of the baseline measurements was assessed using the Shapiro–Wilk test, and between-group differences were evaluated using a one-way ANOVA.

Blinding was not performed because the experimental procedures involved identifiable irrigation protocols, and the outcomes were obtained using standardized mechanical and colorimetric measurements.

The exposure durations shown in Table 1 were selected to reflect the different clinical roles of the irrigants, with NaOCl representing cumulative use during chemomechanical preparation and chelators representing short final-irrigation protocols. The 7% MA and 1% IP6 concentrations were selected based on concentrations previously evaluated as final chelating irrigants [10,12], whereas 5% IP6 was included as a higher experimental concentration to examine potential concentration-associated responses and was not intended to represent an established clinical protocol. Accordingly, the experimental groups represented clinically oriented irrigation protocols rather than equal-duration comparisons of individual solutions. Distilled water served as the control/reference condition, and no positive control was included because the study was designed to compare routinely used and alternative endodontic irrigants with this aqueous reference condition. Each dentin beam was fully immersed in 5 mL of the respective solution in a separate clear polystyrene container. During the specified exposure period, the irrigant was not replenished or agitated; therefore, the procedure represented a standardized static immersion model. The 2.5% sodium hypochlorite and distilled water control groups were kept in their respective solutions for 20 min, whereas the 17% EDTA, 7% MA, 1% IP6, and 5% IP6 groups were kept in their respective solutions for 1 min. The dentin beams were rinsed with distilled water in a 96-well plate using a microplate shaker at 300 rpm to remove residual irrigation solutions from the surface. Then, the post-immersion apparent elastic modulus was determined using the same method as the initial measurement. Following the post-immersion mechanical measurement, the same dentin beams were subjected to the generic colorimetric MMP assay. Sequential assessment of apparent elastic modulus and total MMP activity in the same dentin beams has previously been employed in the literature [24].

### 2.3. Generic Colorimetric MMP Assay

A generic colorimetric MMP assay (SensoLyte Generic MMP Assay, AnaSpec, Fremont, CA, USA; Cat. No. AS-72095) was used to quantify post-treatment total endogenous MMP activity, with mineralized radicular dentin beams serving as the matrix-bound enzyme source. The tissue-beam assay was adapted with modifications from the protocol described by Salim et al. [25].

Following exposure to the irrigation solutions (Table 1), the dentin beams were rinsed, blot-dried with absorbent paper, and individually incubated in 200 μL of freshly prepared chromogenic substrate/assay-buffer working solution in a clear 96-well plate for 120 min at 25 °C. Dentin beams treated with distilled water served as the reference control group. At 30, 60, 90, and 120 min, the beams were temporarily removed from the wells, and absorbance was measured at 412 nm using a microplate reader (Synergy HT, BioTek Instruments, Winooski, VT, USA).

Substrate-control wells containing the substrate/assay-buffer working solution without dentin beams were included to determine background absorbance. The background absorbance recorded at each time point was subtracted from the corresponding specimen readings. In addition, inhibitor-control wells containing dentin beams and the kit-supplied broad-spectrum MMP inhibitor GM6001 were included to verify suppression of the MMP-dependent signal.

The kinetic records showed that the highest observed absorbance occurred at the common 120 min endpoint for all specimens. Therefore, no specimen-specific maximum value was selected, and the background-corrected absorbance measured at 120 min was used for all analyses. Initial reaction velocities were not calculated because the 30 min measurement intervals and the use of solid dentin beams as the matrix-bound enzyme source were intended for a standardized endpoint comparison rather than characterization of the initial linear phase of the reaction.

Background-corrected values were normalized to the mean background-corrected value of the distilled-water control group and expressed as relative total MMP activity using the following equation:

Relative total MMP activity (% of control) = (background-corrected specimen value/mean background-corrected value of the distilled-water control group) × 100.

By definition, this normalization yielded an unrounded mean relative activity of 100% for the distilled-water control group.

### 2.4. Statistical Analysis

Statistical analyses were performed using IBM SPSS Statistics for Windows, version 23.0 (IBM Corp., Armonk, NY, USA). The Shapiro–Wilk test was used to assess normality, and Levene’s test was used to evaluate homogeneity of variances. Group-specific descriptive data were reported as mean ± standard deviation together with two-sided 95% confidence intervals calculated using Student’s t distribution.

Apparent elastic modulus data were analyzed using a two-way mixed ANOVA, with irrigation protocol as the between-subject factor and time as the within-subject factor. Because initial and post-immersion measurements were obtained from the same radicular dentin beam, the irrigation protocol × time interaction was considered the primary outcome. Following a significant interaction, simple-effects comparisons of the estimated marginal means were performed to compare irrigation protocols separately at each measurement time point and to compare initial and post-immersion measurements within each irrigation protocol, with Bonferroni adjustment for multiple comparisons.

Relative total MMP activity values were compared among irrigation protocols using Welch’s ANOVA because the assumption of homogeneity of variance was violated. Tamhane’s T2 test was used for pairwise comparisons, with pairwise mean differences accompanied by Dunn–Šidák-adjusted simultaneous 95% confidence intervals.

The percentage change in apparent elastic modulus was calculated as ΔEM% = [(post-immersion E − initial E)/initial E] × 100. Spearman’s rank correlation coefficient was used to evaluate the pooled bivariate relationship between ΔEM% and relative total MMP activity. To account for treatment-group separation, both variables were rank-transformed and separately residualized against five dummy variables representing the six irrigation protocols. The correlation between the resulting residuals was reported as the group-adjusted partial Spearman rank correlation. Approximate 95% confidence intervals for the pooled and group-adjusted Spearman rank correlation coefficients were calculated using Fisher’s z transformation. For the group-adjusted coefficient, the standard error accounted for the five dummy variables representing the six irrigation protocols. The significance level was set at α = 0.05.

## 3. Results

No specimens were lost during specimen preparation, immersion procedures, mechanical testing, or MMP activity assessment; therefore, all 60 specimens were included in the final analysis.

### 3.1. Apparent Elastic Modulus

The apparent elastic modulus values before and after exposure to the tested irrigation protocols are shown in Figure 2. The group-specific initial and post-immersion apparent elastic modulus values, respectively, were as follows (mean ± standard deviation; 95% confidence interval; *n* = 10 per protocol): control, 3.711 ± 0.399 GPa (3.426–3.996) and 3.705 ± 0.397 GPa (3.421–3.989); 2.5% NaOCl, 3.714 ± 0.384 GPa (3.439–3.988) and 3.692 ± 0.290 GPa (3.485–3.900); 17% EDTA, 3.732 ± 0.368 GPa (3.469–3.995) and 3.666 ± 0.371 GPa (3.400–3.931); 7% MA, 3.733 ± 0.311 GPa (3.511–3.956) and 3.120 ± 0.157 GPa (3.007–3.232); 1% IP6, 3.757 ± 0.299 GPa (3.543–3.971) and 3.738 ± 0.285 GPa (3.534–3.942); and 5% IP6, 3.733 ± 0.271 GPa (3.540–3.927) and 3.175 ± 0.202 GPa (3.030–3.320).

The corresponding ΔEM% values were −0.17 ± 0.08% (95% CI: −0.23 to −0.11) for the control, 0.18 ± 11.41% (95% CI: −7.98 to 8.35) for 2.5% NaOCl, −1.79 ± 1.41% (95% CI: −2.80 to −0.79) for 17% EDTA, −15.97 ± 7.29% (95% CI: −21.18 to −10.76) for 7% MA, −0.45 ± 2.29% (95% CI: −2.09 to 1.19) for 1% IP6, and −14.52 ± 8.56% (95% CI: −20.64 to −8.40) for 5% IP6. These confidence intervals are descriptive and unadjusted; inferential conclusions were based on the Bonferroni-adjusted mixed-ANOVA contrasts reported below.

Baseline apparent elastic modulus values did not differ among groups [F(5,54) = 0.023, *p* = 0.999]. The two-way mixed ANOVA revealed a significant main effect of time [F(1,54) = 42.165, *p* < 0.001, partial η^2^ = 0.438], indicating an overall change in apparent elastic modulus after immersion. The irrigation protocol × time interaction was significant [F(5,54) = 12.817, *p* < 0.001, partial η^2^ = 0.543], indicating that the magnitude of apparent elastic modulus change differed among the tested irrigation protocols. The main effect of irrigation protocol was not statistically significant [F(5,54) = 2.371, *p* = 0.051, partial η^2^ = 0.180]. Bonferroni-adjusted simple-effects comparisons of the estimated marginal means showed no significant differences among irrigation protocols at the initial measurement (all adjusted *p* > 0.05). At the post-immersion measurement, the 7% MA and 5% IP6 protocols resulted in significantly lower apparent elastic modulus values than the control, 2.5% NaOCl, 17% EDTA, and 1% IP6 protocols (adjusted *p* = 0.0003–0.0075). No significant difference was detected between the 7% MA and 5% IP6 protocols or among the other four protocols at the post-immersion measurement (all adjusted *p* > 0.05). Within-protocol comparisons showed significant reductions from initial to post-immersion values following 7% MA and 5% IP6 exposure (both adjusted *p* < 0.001); whereas, no significant changes were detected for the control, 2.5% NaOCl, 17% EDTA, or 1% IP6 protocols (all adjusted *p* > 0.05).

### 3.2. Evaluation of Relative Total MMP Activity of Radicular Dentin

The results of the generic MMP assay are presented in Figure 3 as relative total MMP activity normalized to the mean value of the distilled-water control group. The group-specific relative total MMP activity values were as follows (percentage of control; mean ± standard deviation; 95% confidence interval; *n* = 10 per protocol): control, 100.0 ± 7.2% (94.9–105.1); 2.5% NaOCl, 93.6 ± 4.2% (90.6–96.5); 17% EDTA, 120.2 ± 23.3% (103.5–136.8); 7% MA, 251.8 ± 59.4% (209.3–294.2); 1% IP6, 150.7 ± 20.2% (136.2–165.1); and 5% IP6, 236.5 ± 64.2% (190.6–282.5).

Welch’s ANOVA demonstrated a significant difference in relative total MMP activity among the irrigation protocols [F(5, 23.05) = 36.285, *p* < 0.001]. Tamhane’s T2 pairwise comparisons detected no statistically significant differences among the distilled-water control, 2.5% NaOCl, and 17% EDTA protocols (adjusted *p* = 0.082–0.345). Relative total MMP activity following 1% IP6 exposure was significantly higher than that following distilled water and 2.5% NaOCl exposure (both adjusted *p* < 0.001), but did not differ significantly from that following 17% EDTA exposure (mean difference = 30.4 percentage points; simultaneous 95% CI: −2.5 to 63.3; adjusted *p* = 0.086). Relative total MMP activity following 7% MA exposure was significantly higher than that following distilled water, 2.5% NaOCl, 17% EDTA, and 1% IP6 exposure (adjusted *p* < 0.001, <0.001, <0.001, and =0.005, respectively). Similarly, relative total MMP activity following 5% IP6 exposure was significantly higher than that following distilled water, 2.5% NaOCl, 17% EDTA, and 1% IP6 exposure (adjusted *p* = 0.001, <0.001, =0.003, and =0.031, respectively). No statistically significant difference was detected between the 7% MA and 5% IP6 protocols (mean difference = 15.2 percentage points; simultaneous 95% CI: −77.8 to 108.2; adjusted *p* > 0.999).

### 3.3. Correlation Between Apparent Elastic Modulus Change and Relative Total MMP Activity

Spearman’s rank correlation analysis showed a significant negative pooled association between ΔEM% and relative total MMP activity (r_s_ = −0.679, 95% CI: −0.796 to −0.514, *p* < 0.001). However, after accounting for irrigation protocol as a categorical variable, the group-adjusted association was no longer statistically significant (partial r_s_ = −0.146, 95% CI: −0.396 to 0.124, *p* = 0.288). This attenuation indicates that the pooled association predominantly reflected between-protocol differences rather than a treatment-independent relationship within the irrigation protocols.

## 4. Discussion

Root canal treatment and irrigation procedures may alter the chemical and structural composition of radicular dentin [2,3]. Therefore, new or alternative irrigating solutions should be evaluated not only for smear layer removal or antimicrobial activity but also for their effects on dentin integrity. Limited information is available on the effects of maleic acid (MA) and phytic acid (IP6), which have been proposed as alternatives to EDTA, on the mechanical and enzymatic properties of radicular dentin. Accordingly, the present study evaluated the effects of routinely used root canal irrigants and alternative chelating agents on the apparent elastic modulus and host-derived total MMP activity of radicular dentin.

Apparent elastic modulus and enzymatic activity depend on intrinsic dentin characteristics; therefore, donor- and tissue-related variability should be considered when interpreting the findings. Donor age, sex, and other clinical characteristics were unavailable because anonymous extracted teeth were used, and their potential influence could not be assessed. Although specimen location was standardized by preparing one beam from the coronal radicular dentin adjacent to the canal lumen, dentin composition and mechanical behavior may vary according to root level, anatomical location, tooth type, age, and pathological history. Therefore, the findings should be generalized only to mineralized coronal radicular dentin from single-rooted teeth under the tested laboratory conditions. To limit baseline imbalance related to intrinsic tissue variability, specimens were allocated using a baseline-balanced procedure based on their initial apparent elastic modulus.

The significant irrigation protocol × time interaction indicated that the change in apparent elastic modulus differed among the tested irrigants. Therefore, the first null hypothesis was rejected. In this study, 2.5% NaOCl applied for 20 min did not significantly alter the apparent elastic modulus of radicular dentin, consistent with a previous report using the same concentration and a comparable exposure time [2]. However, most studies reporting deterioration of dentin mechanical properties generally used higher NaOCl concentrations and/or prolonged exposure times [7,26,27,28,29,30,31]. Mechanistically, NaOCl may alter dentin elasticity by penetrating mineralized dentin, degrading collagen, and leaving a collagen-sparse, apatite-rich matrix with reduced mechanical resistance; however, these effects appear to be influenced by both concentration and contact time [7,22]. The limited change in apparent elastic modulus after 2.5% NaOCl exposure may be considered favorable under the tested 20 min condition.

Bonferroni-adjusted within-protocol comparisons showed significant reductions in apparent elastic modulus from initial to post-immersion measurements following 7% MA and 5% IP6 exposure, whereas no significant changes were detected following 17% EDTA or 1% IP6 exposure. Few studies have evaluated the effect of endodontic chelators on the elastic modulus of radicular dentin [26,32,33]. The absence of a significant effect after 17% EDTA exposure is consistent with a previous study [26].

Another previous study reported that both 17% EDTA and 7% MA reduced dentin elasticity [32], which is partly consistent with the present findings. In the present study, EDTA caused only a non-significant reduction, whereas 7% MA significantly affected the apparent elastic modulus of radicular dentin. This difference may be attributed to methodological variations, as the previous study used nanoindentation to assess local mechanical properties of intertubular dentin, whereas the present study used a three-point bending test to evaluate the bulk structural response of dentin beams. Because nanoindentation reflects site-specific mechanical behavior within a limited area, while three-point bending represents the response of the entire specimen, differences in testing scale may account for the significant EDTA-related reduction reported previously but not observed in the present study.

The mechanical response to IP6 differed between the two tested concentration protocols. A slight, non-significant decrease in apparent elastic modulus was observed after 1% IP6 treatment, whereas 5% IP6 produced a significant reduction, indicating a less favorable mechanical response at the higher concentration. Direct comparison with previous studies is limited because the effect of IP6 concentration on the apparent elastic modulus of radicular dentin has not been specifically addressed. Nevertheless, a previous study showed that 2.5% IP6 applied for 5 min did not significantly reduce the flexural strength or surface microhardness of radicular dentin sticks compared with the control [33]. Although flexural strength and microhardness are not directly interchangeable with apparent elastic modulus, they provide complementary information regarding the mechanical behavior of radicular dentin. Therefore, the preservation of dentin mechanical properties reported with 2.5% IP6 may provide indirect support for the present observation that lower IP6 concentrations are more favorable for radicular dentin than 5% IP6. Overall, the significant reductions observed with 5% IP6 and 7% MA, compared with EDTA and 1% IP6, may be associated with their stronger decalcifying and erosive effects on dentin [34].

MMPs are recognized as key mediators in the degradation of the demineralized collagen matrix in coronal dentin. Their activation undermines the organic dentin structure, promotes progressive breakdown of the hybrid layer, and compromises the long-term stability of adhesive bonds. Coronal and root dentin differ in mineral composition, tubule density, permeability, and structural organization [35]. Therefore, understanding how endodontic procedures influence relative total MMP activity in radicular dentin may help optimize adhesive performance and improve long-term outcomes. In the present study, a generic colorimetric assay was used to quantify post-treatment total endogenous MMP activity. The findings showed that relative total MMP activity differed among the tested irrigation protocols; therefore, the second null hypothesis was rejected.

To date, few studies have investigated the effects of endodontic irrigation solutions on post-treatment MMP activity in radicular dentin [21,36,37,38]. In the present study, relative total MMP activity was significantly higher in the 5% IP6 and 7% MA groups than in the control group, whereas neither the 17% EDTA protocol applied for 1 min nor the 2.5% NaOCl protocol applied for 20 min differed significantly from the control. In contrast to our findings, two dentin-specific studies reported reduced MMP activity following higher-concentration NaOCl protocols (5–5.25%), whereas chelating protocols involving 18% EDTA or 10% citric acid were associated with increased MMP activity following dentin demineralization [21,36]. In another study, it was reported that 17% EDTA applied for 1 min to completely demineralized dentin resulted in lower MMP activity [38]. Differences in methodology, such as test method, specimen preparation, irrigation solution concentration, and application time, may partially explain the differences in findings across studies. Zymographic analysis using powdered dentin can identify specific MMP isoforms but disrupts the dentin matrix, potentially exaggerating enzyme release. In contrast, the present study assessed total MMP activity colorimetrically in intact mineralized dentin beams, thereby preserving tissue organization and representing the mineralized radicular dentin matrix more closely than the powdered-dentin model. Another methodological difference is the demineralization process. Prolonged or repeated EDTA application during demineralization has been reported to increase MMP activity at the mineral–organic interface within the dentinal tubules [21]. Therefore, our protocol did not include multiple EDTA demineralization cycles, as we aimed to assess its effect as an endodontic irrigation solution. In another study evaluating the effects of the final irrigation protocol on DMP1-CT expression, a marker associated with relative total MMP activity, the sequential application of 2.5% NaOCl and 18% EDTA resulted in MMP activity similar to that observed with distilled water, as in our study [37].

To the best of the authors’ knowledge, no previous study has evaluated the effects of IP6 or MA on total MMP activity in radicular dentin. In the present study, the 1% IP6, 5% IP6, and 7% MA groups exhibited significantly higher relative total MMP activity than the distilled-water control and NaOCl groups. No statistically significant difference in relative total MMP activity was detected between the 1% IP6 and 17% EDTA groups, whereas the 5% IP6 and 7% MA groups exhibited significantly higher relative total MMP activity. This group-dependent pattern may be associated with differences in pH and demineralizing capacity among the tested irrigation protocols. However, because only post-treatment total MMP activity was measured, the assay cannot determine whether the observed differences resulted from activation of latent MMPs, altered accessibility of matrix-bound enzymes, or other treatment-related effects. In addition, acidic conditions may destabilize MMP propeptides, increasing their likelihood of activation [2,3].

Because no previous study has evaluated the effect of IP6 on MMP activity in mineralized radicular dentin beams, direct comparison with the present findings is limited. Studies using demineralized coronal dentin have reported no statistically significant difference in total MMP activity between IP6-treated specimens and control specimens, regardless of the IP6 concentration tested (1–3%) [39,40]. This absence of elevated activity has been attributed to the ability of phytic acid to interact with dentin collagen, chelate calcium and zinc ions required for MMP catalytic activity and stability, or interact directly with the enzymes [39,40]. However, demineralized coronal dentin differs from the mineralized radicular dentin beams used in the present study, particularly in mineral content, exposed collagen-associated functional groups, and available binding sites for phytic acid. Thus, the use of mineralized radicular dentin beams may provide a substrate more representative of mineralized radicular dentin for evaluating irrigant-induced MMP activity and may partly explain the differences between the present findings and those reported in demineralized coronal dentin.

The pooled analysis showed a significant negative association between ΔEM% and relative total MMP activity; therefore, the third null hypothesis was rejected at the pooled-analysis level. However, this association was no longer statistically significant after adjustment for irrigation protocol. The marked attenuation of the association indicates that the pooled relationship predominantly reflected between-protocol differences. In particular, the 7% MA and 5% IP6 protocols exhibited both the greatest reductions in apparent elastic modulus and the highest measured post-treatment total MMP activity. Thus, these outcomes changed concurrently under the tested protocols, but the findings do not demonstrate a mechanobiological relationship between mechanical change and total MMP activity independent of irrigation protocol. These results emphasize the importance of considering both mechanical and enzymatic responses when evaluating the effects of chelating irrigation protocols on radicular dentin.

An important strength of the present study is the combined evaluation of mechanical and enzymatic responses in mineralized radicular dentin beams. The use of intact dentin beams preserved the tissue’s structural organization more closely than powdered or completely demineralized dentin models, allowing the effects of irrigants to be assessed in a substrate more representative of mineralized radicular dentin. In addition, baseline-balanced allocation limited between-group imbalance in initial apparent elastic modulus and supported the interpretation of post-immersion changes.

The generic colorimetric MMP assay measured total activity across a broad range of MMPs but could not distinguish between individual MMP types or isoforms [41]. Although three-point bending to 3% strain was selected as a non-destructive testing protocol, a separate unloaded control group was not included; therefore, a subtle influence of repeated mechanical loading on the subsequent MMP measurements cannot be completely excluded. In addition, exposure durations were selected to reflect the different clinical roles of the irrigants and therefore differed among protocols, preventing the independent effects of solution type and exposure duration from being distinguished. Moreover, the standardized static immersion of one dentin beam in 5 mL of irrigant did not reproduce the dynamic conditions of clinical root-canal irrigation, including continuous irrigant flow, replenishment, irrigant activation, and the anatomical constraints of the root-canal system. Accordingly, the findings should be interpreted as laboratory protocol-dependent effects and should not be directly extrapolated to clinical irrigation. The identical exposure durations used for 1% and 5% IP6 permit a direct comparison between these protocols; however, IP6 concentration and pH varied concurrently and their individual contributions could not be distinguished. MMP activity was also evaluated at a single post-treatment endpoint, precluding assessment of temporal changes. Future studies should identify the relevant MMP isoforms and examine equal-duration comparisons, sequential irrigation protocols, dynamic irrigation models, and longer-term enzymatic effects.

## 5. Conclusions

Within the limitations of this in vitro study, protocol-dependent differences were observed in the mechanical response and post-treatment relative total MMP activity of mineralized radicular dentin. The less favorable mechanical and enzymatic profiles observed with 7% MA and 5% IP6 underscore the importance of evaluating alternative chelating protocols not only for their cleaning efficacy but also for their effects on dentin integrity. Low-concentration IP6 warrants further investigation as a potential chelating alternative to EDTA; however, its equivalence to or ability to substitute for EDTA cannot be established from the mechanical and enzymatic outcomes evaluated in the present study. Further studies addressing cleaning efficacy, antimicrobial activity, cytotoxicity, bonding performance, and long-term effects are required.

## Figures and Tables

**Figure 1:**
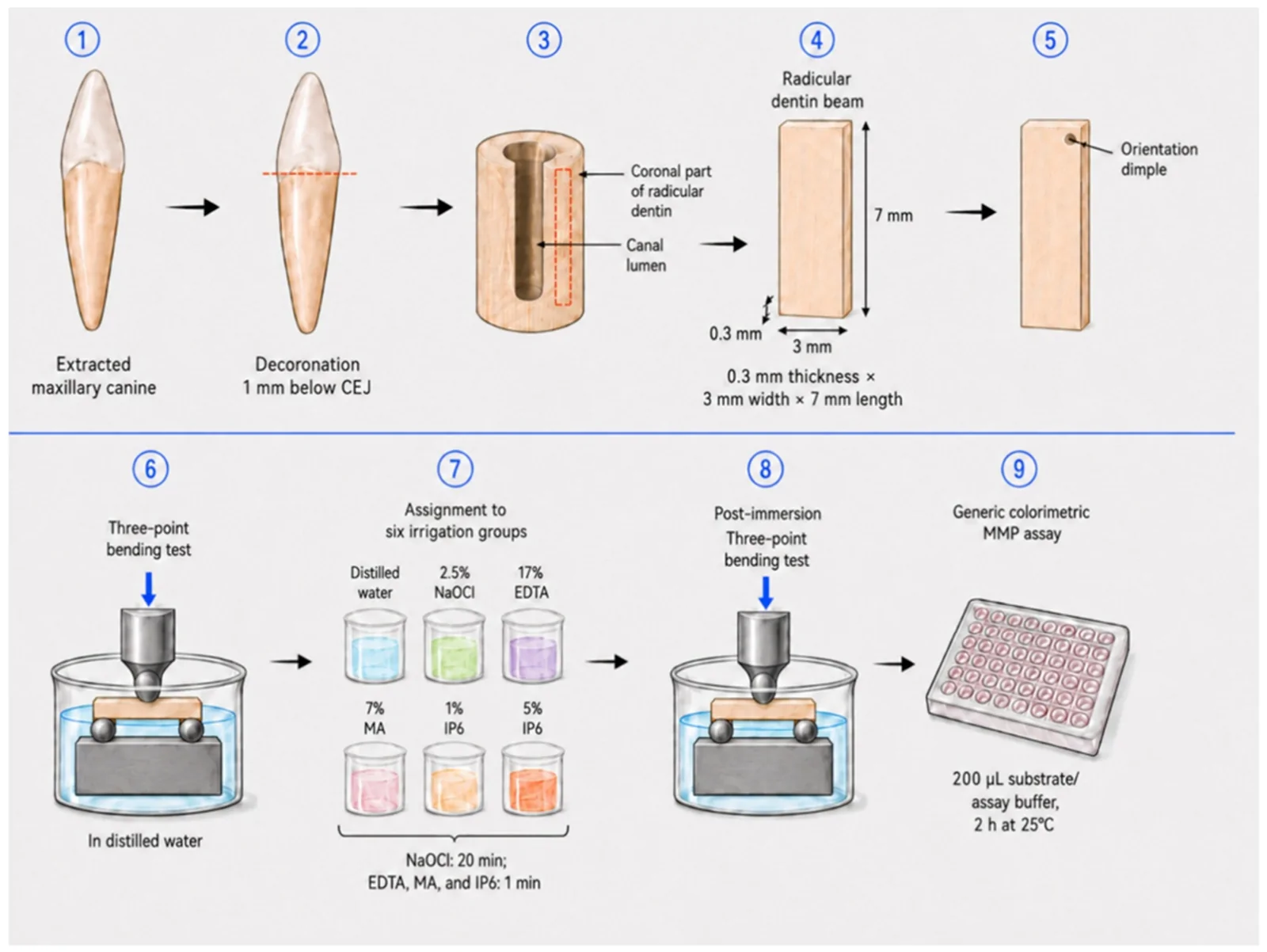
Schematic representation of radicular dentin beam preparation and the experimental workflow. Black arrows indicate the sequence of experimental steps; red dashed markings indicate the decoronation level and the location of beam preparation; blue arrows indicate the direction of loading during three-point bending.

**Figure 2:**
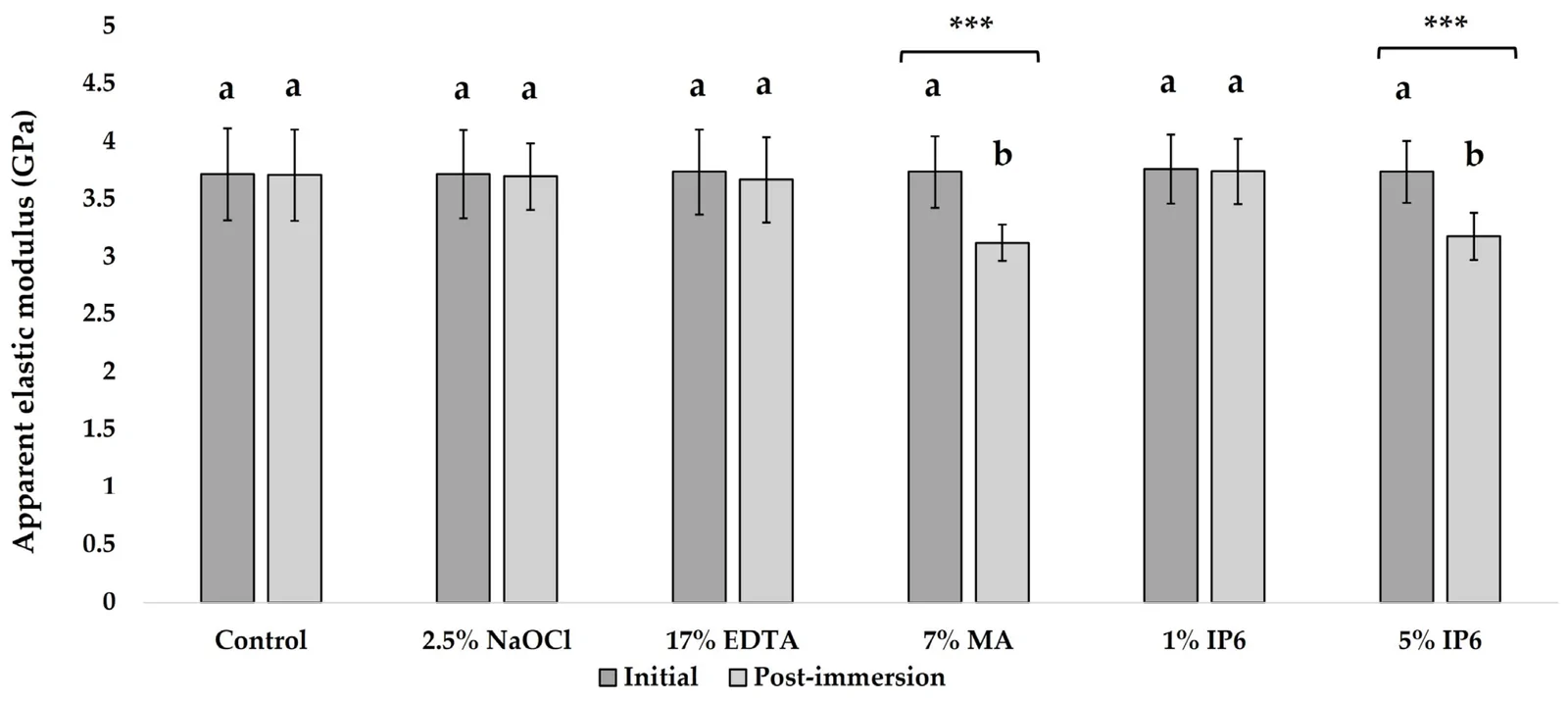
Apparent elastic modulus values of radicular dentin before and after immersion in the tested irrigation protocols. Bars represent mean ± standard deviation. Initial and post-immersion measurements were obtained from the same radicular dentin beams. Within each measurement time point, bars with different lowercase letters indicate statistically significant differences among irrigation protocols according to Bonferroni-adjusted simple-effects comparisons of the estimated marginal means (*p* < 0.05); bars sharing the same letter did not differ significantly. Brackets with asterisks indicate significant differences between initial and post-immersion measurements within the same irrigation protocol (*** *p* < 0.001).

**Figure 3:**
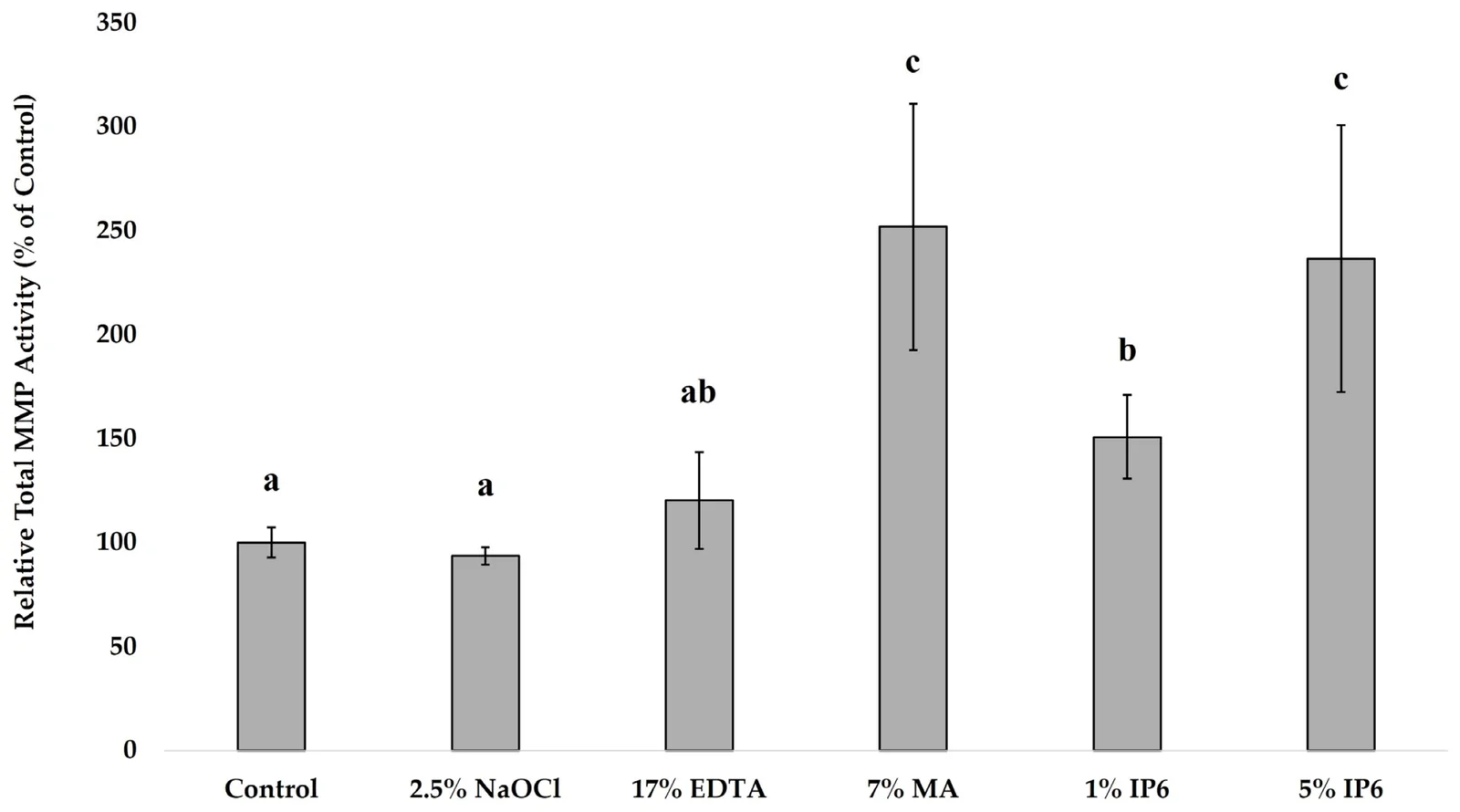
Relative total endogenous MMP activity in mineralized radicular dentin following exposure to the tested irrigation protocols. Values were normalized to the mean value of the distilled-water control group, which was set at 100%. Bars represent mean ± standard deviation. Different lowercase letters indicate statistically significant differences among protocols according to Tamhane’s T2 pairwise comparisons (*p* < 0.05).

**Table 1:** Irrigation solutions used for the treatment of radicular dentin beams.

Group	Solution	pH *	Concentration	Application Time
Control	Distilled water	7.0		20 min
NaOCl	Sodium hypochlorite	12	2.5%	20 min
EDTA	Ethylenediaminetetraacetic acid	7.4	17%	1 min
MA	Maleic acid	1.8	7%	1 min
1% IP6	Phytic acid	1.15	1%	1 min
5% IP6	Phytic acid	0.7	5%	1 min

* All irrigation solutions were freshly prepared immediately before use, and their pH values were measured before specimen immersion using the same pH meter under identical room-temperature laboratory conditions.

## Data Availability

The raw data supporting the conclusions of this article will be made available by the authors on request.
